# Plasma-Derived Extracellular Vesicles Circular RNAs Serve as Biomarkers for Breast Cancer Diagnosis

**DOI:** 10.3389/fonc.2021.752651

**Published:** 2021-11-10

**Authors:** Li Lin, Geng-Xi Cai, Xiang-Ming Zhai, Xue-Xi Yang, Min Li, Kun Li, Chun-Lian Zhou, Tian-Cai Liu, Bo-Wei Han, Zi-Jia Liu, Mei-Qi Chen, Guo-Lin Ye, Ying-Song Wu, Zhi-Wei Guo

**Affiliations:** ^1^ Key Laboratory of Antibody Engineering of Guangdong Higher Education Institutes, School of Laboratory Medical and Biotechnology, Southern Medical University, Guangzhou, China; ^2^ Department of Breast Surgery, The First People’s Hospital of Foshan, Foshan, China; ^3^ Sun Yat-Sen Memorial Hospital, Sun Yat-Sen University, Guangzhou, China; ^4^ Department of Cancer Biology, Guangzhou XGene Co., Ltd., Guangzhou, China

**Keywords:** breast cancer, cancer diagnosis, extracellular vesicles, circular RNA, predictive classifier

## Abstract

Breast cancer is the second cause of cancer-associated death among women and seriously endangers women’s health. Therefore, early identification of breast cancer would be beneficial to women’s health. At present, circular RNA (circRNA) not only exists in the extracellular vesicles (EVs) in plasma, but also presents distinct patterns under different physiological and pathological conditions. Therefore, we assume that circRNA could be used for early diagnosis of breast cancer. Here, we developed classifiers for breast cancer diagnosis that relied on 259 samples, including 144 breast cancer patients and 115 controls. In the discovery stage, we compared the genome-wide long RNA profiles of EVs in patients with breast cancer (n=14) and benign breast (n=6). To further verify its potential in early diagnosis of breast cancer, we prospectively collected plasma samples from 259 individuals before treatment, including 144 breast cancer patients and 115 controls. Finally, we developed and verified the predictive classifies based on their circRNA expression profiles of plasma EVs by using multiple machine learning models. By comparing their circRNA profiles, we found 439 circRNAs with significantly different levels between cancer patients and controls. Considering the cost and practicability of the test, we selected 20 candidate circRNAs with elevated levels and detected their levels by quantitative real-time polymerase chain reaction. In the training cohort, we found that BC_ExoC_, a nine-circRNA combined classifier with SVM model, achieved the largest AUC of 0.83 [95% CI 0.77-0.88]. In the validation cohort, the predictive efficacy of the classifier achieved 0.80 [0.71-0.89]. Our work reveals the application prospect of circRNAs in plasma EVs as non-invasive liquid biopsies in the diagnosis and management of breast cancer.

## Introduction

Breast cancer is a major kind of malignant tumor that seriously endangers women’s health. According to cancer statistics in 2018, breast cancer accounts for more than 10% of all new diagnoses and causes about 600,000 deaths every year (1). Although the overall prognosis of breast cancer is good, the five-year relative survival rate of stage IV patients is still lower than 30% (2). Early diagnosis of cancer could effectively improve their therapeutic effects. Therefore, it is necessary to develop an early diagnosis method for breast cancer identification.

Plasma extracellular vesicles (EVs), such as exosomes and microvesicles, are mainly derived from cancer and hematopoietic cells in cancer patients, which host cell-information of their original tissues (3, 4). Since the contents of EVs could reflect the characteristics of cancer cells, they have been used to develop a variety of non-invasive methods for cancer-related applications, such as early diagnosis and prognosis prediction of cancer (5–8). For example, microRNAs and proteins derived from EVs have been used for the early diagnosis of various cancers (5, 9, 10). However, instability of microRNA and low abundance of proteins may limit their clinical applications. Therefore, a stable biomarker with appropriate concentrations may be more suitable for the early diagnosis of breast cancer.

Circular RNA (circRNA) is a new type of RNA, which shows the remarkable feature of being covalently closed continuous loops without 5’ to 3’ polar structure (11). CircRNA is stable, temporospatial (often exhibit type-specific, tissue-specific, and stage-specific manner), and conserved (12, 13). Functional studies have shown that they may play important roles in tumorigenesis by becoming microRNA sponge or translating into proteins (14, 15). Recently, a variety of RNAs, especially circRNAs, were discovered in EVs of different types of cancer (16, 17). Since circRNA is stable and type-specific, we assume that the circRNA in the EVs can be used for early diagnosis of breast cancer.

In this study, we first implemented genome-wide long RNA sequencing to determine the difference of RNA profiles in EVs of plasma between breast cancer patients and controls. In the training stage, the circRNAs with significantly different levels were selected and their relative levels were evaluated among 182 participants by quantitative real-time polymerase chain reaction (qPCR). Based on the relative levels, we then constructed the diagnosis classifier with multiple machine learning models, including SVM, LR, and LDA. According to the results of their cross-validation, the classifier with the largest AUC was selected, and its performance was further studied in the validation cohort.

## Methods

### Participants and Research Design

**Figure 1:**
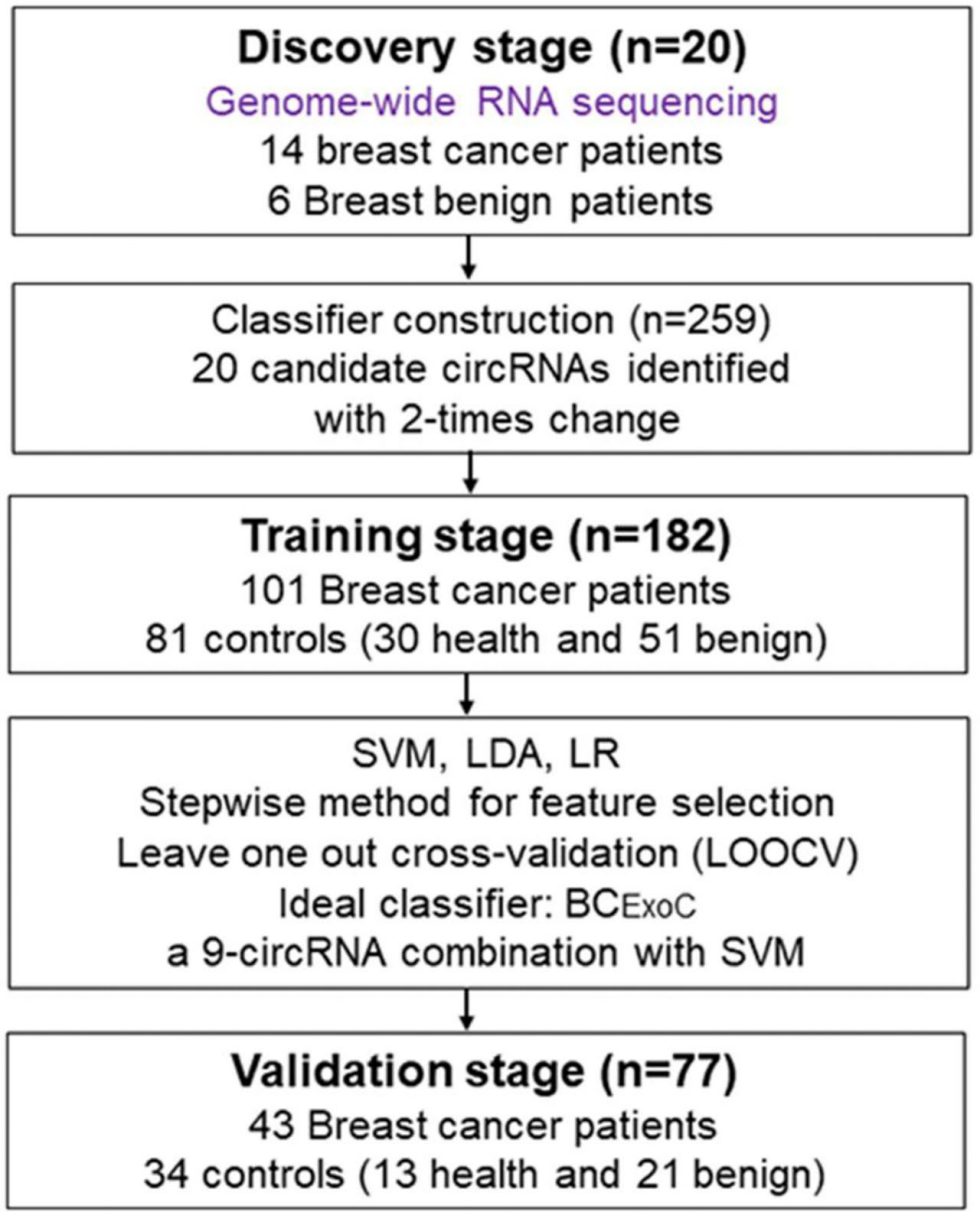
Study design. To develop classifiers for the early diagnosis of breast cancer, the workflow of our study consists of three stages, including the discovery stage, training, and validation stage. In the discovery stage, we used whole-genome sequencing to identify circRNAs with significantly different levels. In the training stage, we developed classifiers with three regression models by using the circRNAs levels detected by qPCR. In the validation stage, the predictive efficacy of the classifiers was validated. qPCR, quantitative real-time polymerase chain reaction. circRNA, circular RNA.

In total, we collected 259 plasma samples from two groups of individuals: breast cancer patients and controls (including healthy individuals and benign breast patients including fibroadenoma and benign epithelial proliferation, **Figure 1**). Participants were enrolled from the First People’s Hospital of Foshan and plasma samples of all cancer patients were collected prospectively before cancer therapy. The samples used in the discovery stage were collected prospectively from January 2018 to July 2018. Plasma samples used in the training and validation stage were collected prospectively from August 2018 to May 2019. All plasma samples were obtained under institutional review board of the First People’s Hospital of Foshan approved protocols with written informed consent from all participants for research use [ID: L(2021)-7]. More details about the clinical information of all participants involved in this study were shown in **Supplemental Table 1**.

### RNA Extraction and Sequencing

RNAs of EVs were extracted from about 5 mL of plasma with exoRNeasy Serum/Plasma Maxi Kit following the manufacturer’s instructions (QIAGEN, Germany). In brief, the plasma was prefiltered, then was mixed with 2x binding buffer. The mixture is added to the exoEasy membrane affinity to bind the EVs to the membrane. After centrifugation, the wash buffer was added to wash off non-specific material in the column. After enriching EVs, QIAZOL was added to the column to lyse the vesicles and chloroform was added to the lysate collected after centrifugation. After the aqueous phase is recovered and mixed with ethanol, and the sample-ethanol mixture is added to the RNeasy MinElute spin column and centrifuged. Washing the column with buffer RWT, then wash twice with buffer RPE. And finally elute RNA in water. The rRNAs in total RNAs were first removed using Ribo-Zero rRNA Removal Kits (Illumina, USA) and the libraries of RNA-sequencing were constructed with TruSeq Stranded Total RNA Library Prep Kit (Illumina, USA). Subsequently, quality and quantification of libraries were assessed using the BioAnalyzer 2100 system (Agilent Technologies, USA). Finally, 10 pM libraries were denatured as single-stranded DNA molecules, captured on Illumina flow cells, amplified *in situ* as clusters, and finally sequenced for 150 cycles on Illumina HiSeq 4000 sequencer following the manufacturer’s instructions.

### Process of High Throughput RNA-Sequencing Data

The 3’ adapter of the raw read was trimmed, and the low-quality read was removed by using cutadapt software (v1.9.3). At first, the reads were aligned to the reference genome (hg19) and transcriptome with STAR software (v2.5.1b) (18). Then, the circRNAs were detected and identified by DCC software (v0.4.4) (19). According to their genomic localization of known genes, the circRNAs were separated into five different types, including exon, intronic, intergenic, antisense, and sense overlapping circRNAs. In addition, the identified circRNAs were annotated with circBase and some previous studies (20–22). Normalized expression values of circRNAs were calculated by using edgeR software (v3.16.5) (23). For LncRNA and mRNA, the reads were aligned to the human reference genome with hisat2 software (v2.0.4) (24).

The dysregulated circRNAs were determined by the edgeR package of R software with a cutoff threshold of |log_2_ fold change| ≥ 2 and *P*-value < 0.05 (**Supplemental Table 2**). The principal component analysis (PCA) and result visualization were realized by rgl package (v0.1). The enrichment of GO function and KEGG pathway were implemented and visualized by using Metascape (25) and OmicShare tools (www.omicshare.com/tools).

### Detection of qPCR

TaKaRa PrimeScript™ RT reagent was done with equal quality of input RNA. The qPCR for human circRNAs was done on an Applied Biosystems 7500 Real-Time PCR System using the TaKaRa TB Green™ Premix Ex Taq™ II. The value of the cycle threshold (Ct) was processed and exported by the software of Applied Biosystems SDS (v2.3.0). CircRNAs from the training and testing cohort were detected by qPCR with a human endogenous mRNA, U6, as a reference. Relative quantification was used and the levels of circRNAs were normalized against the level of reference by 2^–Δct^, where ΔCt = Ct_target_ – Ct_reference_. Their primer sequence was shown in **Supplemental Table 3**.

### Construction of Classifiers for Early Diagnosis of Breast Cancer

The workflow of classifier construction was shown in **Figure 1**. Firstly, the circRNAs with significantly elevated levels in breast cancer patients were selected. In the training stage, the relative level of 20 candidate circRNAs in 182 participants, including 101 breast cancer patients and 81 controls (30 healthy individuals and 51 breast benign patients [42 fibroadenoma and 9 benign epithelial proliferation]), was assessed using qPCR (**Figure 1**). To construct circRNA classifiers that could distinguish breast cancer patients from controls, the qPCR was used to develop classifiers with three regression modes, including support vector machine (SVM), logistic regression (LR), and linear discriminate analysis (LDA). The SVM classifier was constructed with the linear kernel in e1071 package using the default setting. The glm and lda function in base package of R software was used to develop the LR and LDA classifier with default setting, respectively.

Since quite a number of studies have reported that discrete data may improve classifier performance (26), before classifier construction, the continuous variable was first discretized according to the optimal cut-off point. The optimal cut-off point of each variable was defined as the maximum value of (sensitivity + specificity)/2 in the training cohort. Then the continuous value set to one when it was larger than the corresponding optimal cut-off in each subject; Otherwise, it was set to zero (**Supplemental Table 4**). The stepwise method was used to select the optimal classifier with the largest AUC. To estimate the robustness and prediction error of the selected classifiers, we applied the leave one out cross-validation (LOOCV) method. Briefly, each subject in the training cohort was withheld in turn, and the rest of subjects were submitted to train the model. As there were 182 samples in the training cohort, this procedure was repeated 182 times. In the validation cohort, the relative levels of the circRNAs in the selected classifiers were detected, which included 77 participants, including 43 breast cancer patients and 34 controls [13 healthy individuals and 21 breast benign patients (17 fibroadenoma and 4 benign epithelial proliferation)]. Finally, the predictive efficacy of the optimal classifier in the validation cohort was calculated.

### Statistical Analysis

The fisher exact test and the χ^2^ test were used for comparison of categorical variables. *P-*value < 0.05 for two-sided tests was considered to be statistically significant. Hierarchical clustering was applied to the circRNAs with significantly different levels, using the average‐linkage clustering algorithms in Cluster (ver. 3.0). Heat maps were plotted using the pheatmap package of R (version 3.0.1). The receiver operating characteristic curve (ROC) was drawn and the difference of the area under the curve (AUC) was calculated by using the pROC package (27).

## Results

### Genome-Wide Long RNA Profiles of Plasma EVs

**Figure 2:**
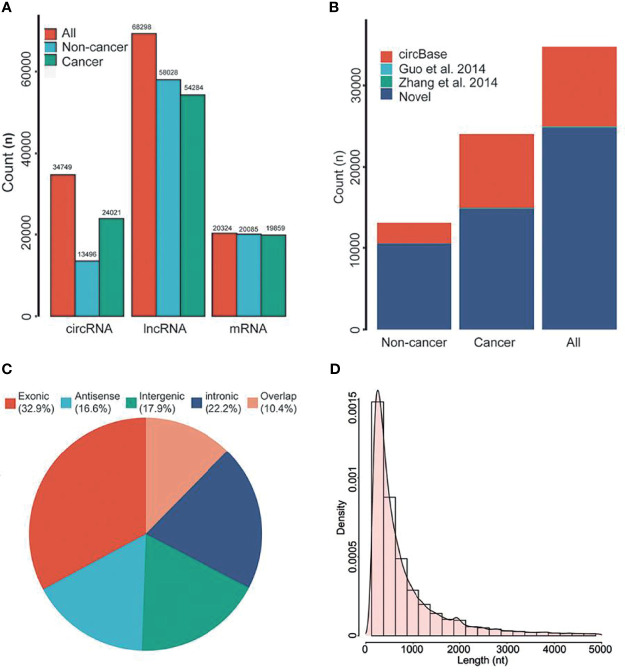
RNA composition in EVs. **(A)** The types of RNAs in EVs. **(B)** Annotation of circRNAs. **(C)** Source of circRNAs. **(D)** Length distribution of circRNAs. nt, Nucleotide. All, all of individuals. Overlap, sense overlapping circRNAs.

Previous studies have shown that a variety of RNA was found in EVs of plasma. Therefore, we first analyzed the genome-wide long RNA profiles of breast cancer patients and controls. We found that there existed different types of RNAs in EV, such as circRNA, lncRNA, and mRNA, and each type of RNA showed many entities [circRNA (n=34,749), lncRNA (n=68,298) and mRNA (n=20,324); **Figure 2A**]. The amount of circRNAs derived from breast cancer patients was significantly higher than that of benign patients (**Figure 2A**). However, this phenomenon has not been observed in lncRNA and mRNA. In addition, the circular structure of circRNAs was normally more stable than the linear RNA, so they may be suitable to be disease biomarkers.

By using public databases and literatures to annotate the circRNAs, we found that approximately 71.30% of circRNAs were novel circRNAs (**Figure 2B**). According to previous classification criteria, we characterized the circRNAs into five types, including exonic, intronic, intergenic, sense overlapping and antisense circRNAs. We found that the exonic and intronic circRNAs took up the largest proportion of circRNAs (55.1%, **Figure 2C**). By further analyzing their length distribution, we found that the majority of circRNAs in EVs were less than 2,500 nucleotides (nt), which took up over 85.92% (**Figure 2D**).

### Distinct CircRNA Profiles Between Breast Cancer Patients and Controls

**Figure 3:**
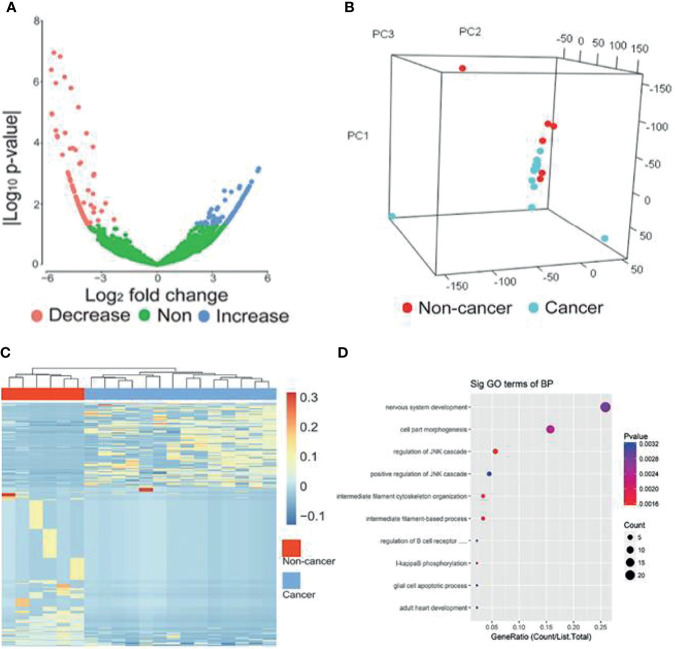
circRNAs with significantly different levels. **(A)** Volcano plots of circRNAs with significant different levels (|log2 fold change| ≥ 2 and *P*-value < 0.05 produced by edgR package of R software) between cancer and control groups. **(B)** PCA analysis of genome-wide RNA sequencing data derived from 14 breast cancer patients and 6 benign patients. **(C)** Heat map of the z-scores of circRNAs with significantly different levels. **(D)** Gene function enrichment analysis of the host genes of the circRNAs with significantly different levels. Decrease, circRNAs with decreased levels. Non, circRNAs with non-significant changes. Increase, circRNAs increased levels.

The workflow of classifier construction was shown in **Figure 1**. In the discovery stage, by comparing the circRNA profiles of 14 breast cancer and 6 benign patients, we identified 439 circRNAs of EVs with significantly different levels, including 162 increased and 277 decreased circRNAs, and the cut-off threshold (|log_2_ fold change|>2, *P*-value<0.05) was calculated by edgR (**Figure 3** **A**, **Supplemental Table 2**). The PCA results showed that the expression profiles between cancer patients and controls showed different patterns (**Figure 3B**). There is approximately 64.69% of circRNAs were novel circRNAs and the exonic and intronic type took up 54.44% among the dysregulated circRNAs. Next, we implemented unsupervised cluster analysis, and found that there was a distinct pattern between the cancer patients and controls (**Figure 3C**), which indicated that circRNAs in EVs may be used for the diagnosis of breast cancer.

To further reveal the relationships between the dysregulated circRNAs and breast cancer, we implemented gene function enrichment analysis on the host genes of circRNAs. The results showed that these terms were enriched in multiple processes, such as cell part morphogenesis, regulation of the JNK pathway and I-kappaB phosphorylation (**Figure 3D**). Previous studies have reported that the enriched pathways were related to the tumorigenesis of breast cancer. For example, the JNK pathway influences proliferation, differentiation, survival and migration in different cancers (28).

### Classifiers for Early Diagnosis of Breast Cancer

**Figure 4:**
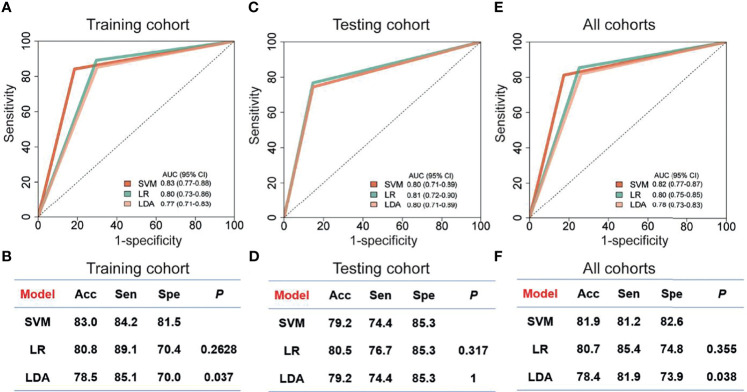
Performance of classifiers for breast cancer prediction. The performance of training cohort **(A, B)**, testing cohort **(C, D)** and all sample **(E, F)** with three models was showed. The AUC in the training cohort was cross-validated using leave one out cross-validation (LOOCV). Receiver operating characteristic, ROC; Acc, accuracy; Sen, sensitivity; SVM, support vector machine; LDA, linear discriminate analysis; LR, logistic regression; Spe, specificity; P, the *P*-value of DeLong's test.

Since the up-regulated features were more practical in clinical detection, we focused on the 20 circRNAs, which were increased in breast cancer patients compared to controls (**Supplemental Figure 1**). In the training cohort, we evaluated the relative levels of 20 increased circRNAs in 182 plasma samples, including 101 breast cancer patients and 81 controls. Three regression models, including SVM, LDA and LR, were used to construct circRNA classifiers which could distinguish breast cancer patients from controls. The AUC, accuracy, sensitivity and specificity of the classifiers were cross-verified by LOOCV cross-validation method (**Figure 4**). Among all combinations with three different regression models, a nine-circRNA combination, named BC_ExoC_, achieved high performance [AUC=0.83 (95% confidence interval 0.77-0.88) and accuracy=0.83] in the training cohort after LOOCV, displaying the maximum AUC of SVM model (**Figures 4A,B**). The circRNAs in BC_ExoC_ were hsa_circ_0002190, hsa_circ_0007177, hsa_circ_0000642, hsa_circ_0001439, hsa_circ_0001417, hsa_circ_0005552, hsa_circ_0001073, hsa_circ_0000267, and hsa_circ_0006404 (**Supplemental Table 4**).

Then, the performance of BC_ExoC_ was investigated and verified in the validation cohort. In the validation cohort, the AUC of BC_ExoC_ was 0.80 (95% CI 0.71-0.89, **Figures 4C, D**). By contrast with the training cohort, the AUC of the validation cohort was similar to those in the training cohort (*P*-value= 0.582; DeLong’s test).

## Discussion

By analyzing genome-wide long RNA sequencing data of EVs, we identified a large number of novel circRNAs in human blood. By comparing the levels between breast cancer patients and those of controls, we found 439 circRNAs with significantly different levels. Based on their levels in EVs, we developed classifiers with three regression models in the training cohort. The optimal classifier (BC_ExoC_) composed of nine circRNAs with the highest AUC was selected, and then it was verified in the validation cohort. The AUC of BC_ExoC_ was 0.82 [0.77-0.87] in all cohorts (**Figures 4E, F**). These findings highlight the potential of BC_ExoC_ as a non-invasive assessment for breast cancer in preclinical stages.

Compared with other studies, our method has several strengths: The circRNAs showed temporospatial characteristics (exhibit patient-specific and stage-specific manner), and the heat map results showed distinct patterns between breast cancer patients and controls, indicating their potentials as early diagnostic biomarkers of breast cancer. In this study, we used circRNAs in plasma EVs, so our method is non-invasive, which could reduce the harm of biopsy to patients and avoid the heterogeneity of cancer. In addition, the circular structure of circRNAs was normally more stable than the linear RNA, therefore, they may be more suitable taken as disease biomarkers. However, our study also has some limitations: as all samples were merely collected from one center, the performance of our classifier needs to be validated with more independent cohorts prior to their clinical applications.

By literature search, we found that three of the top 20 up-regulated circRNAs have been studied in breast cancer cells, and their functions were closely related to tumorigenesis (29, 30). For example, silencing of has_circ_0004771 inhibits proliferation and induces apoptosis in breast cancer through activation of miR-653 by targeting ZEB2 signaling pathway (29). Results of gene function enrichment analysis showed that some of these circRNAs were related to the tumorigenesis of breast cancer. Searching for the functions of these genes is expected to be biomarkers or therapeutic targets for breast cancer.

In summary, our data showed that BC_ExoC_ is a promising noninvasive method for the early diagnosis of breast cancer. Our techniques required for circRNA detection, such as plasma collection, RNA extraction, and qPCR, are routinely used in clinics. What’s more, the cost of reagents and consumables is relatively low and the result of BC_ExoC_ is easy to be explained. Therefore, it is feasible to analyze BC_ExoC_ in clinical practice.

## Data Availability Statement

The datasets presented in this study can be found in online repositories. The names of the repository/repositories and accession number(s) can be found in the article/**Supplementary Material**.

## Ethics Statement

The studies involving human participants were reviewed and approved by Institutional review board of The First People’s Hospital of Foshan. The patients/participants provided their written informed consent to participate in this study.

## Author Contributions

G-lY, Y-sW designed and supervised the study. LL and Z-wG analyzed and interpreted the data and prepared the manuscript. X-mZ, LL, B-w H, Z-j L and M-qC designed the study, provided samples and interpreted clinical data. X-xY, ML, KL, C-lZ and T-cL analyzed and interpreted the data. All authors contributed to the article and approved the submitted version.

## Funding

The work was supported by National Natural Science Foundation of China (81872416, 82173001, 81802435, 81900191), Medical Scientific Research Foundation of Guangdong Province of China (B2017006), and China Postdoctoral Science Foundation funded project (2019M662998) and Special fund of Foshan Summit plan (2020G010).

## Conflict of Interest

Author KL was employed by Guangzhou XGene Co., Ltd.

The remaining authors declare that the research was conducted in the absence of any commercial or financial relationships that could be construed as a potential conflict of interest.

The reviewer LZ declared a shared affiliation, with no collaboration, with the authors to the handling editor at the time of the review

## Publisher’s Note

All claims expressed in this article are solely those of the authors and do not necessarily represent those of their affiliated organizations, or those of the publisher, the editors and the reviewers. Any product that may be evaluated in this article, or claim that may be made by its manufacturer, is not guaranteed or endorsed by the publisher.
