# Supplementary material for: Plasma-Derived Extracellular Vesicles Circular RNAs Serve as Biomarkers for Breast Cancer Diagnosis
Source: Front Oncol. 2021 Nov 10;11:752651. doi: 10.3389/fonc.2021.752651 (PMC8660094; doi:10.3389/fonc.2021.752651)
Supplement: Supplementary file 1 [file DataSheet_1.pdf]

Supplemental Materials

Supplementary Table, Figure and Repositories
Supplemental Table 1. Clinical characteristics of the patients and control individuals in this study
Supplemental Table 2. circRNAs with significantly different levels.
Supplemental Table 3. Primer of the candidate circRNA.
Supplemental Table 4. The information of the candidate circRNAs
Supplemental Figure 1. The relative levels of 20 selected circRNAs
Data Repositories

Supplemental Table 1. Clinical characteristics of the patients and control individuals in this study

	Tumor (n=144)	Benign (n=72)	Healthy (n=43)
Age			
<40	27 (18.75%)	45 (62.5%)	25 (58.1%)
$\geq$40	117 (71.25%)	27 (37.5%)	18 (41.9%)
TNM stage			
0	16 (11.11%)		
I	41 (28.47%)		
II	70 (48.61%)		
III	14 (9.72%)		
IV	3 (2.09%)		
Molecular Subtype			
ER/PR+ Her2+	94 (65.28%)		
ER/PR+ Her2-	19 (13.19%)		
ER/PR- Her2+	26 (18.06%)		
ER/PR- Her-	5 (3.47%)		

Data are n, n (%). ER= estrogen receptor. PR= progesterone receptor. Her2= human epidermal growth factor receptor-2.

Supplemental Table 2. circRNAs with significantly different levels.

CircRNAID	logFC	logCPM	PValue	Source	Type	length
chr6:31238920-31324013-	6.03	9.75	1.71E-04	novel	SO	85094
chr19:6702138-6702590-	5.53	9.37	6.87E-04	circBase	exonic	195
chr21:16386665-16415895-	5.46	9.32	8.16E-04	circBase	exonic	203
chr19:13039156-13039661-	5.14	9.12	1.82E-03	circBase	exonic	338
chr18:8718422-8720494+	5.13	9.11	1.86E-03	circBase	exonic	384
chr10:5836848-5842668-	5.01	9.04	2.55E-03	circBase	exonic	343
chr2:168920010-168986268-	4.48	9.64	2.61E-03	circBase	exonic	402
chr9:37424842-37426651+	4.46	9.52	3.02E-03	circBase	exonic	321
chr14:34394824-34400421-	4.95	9.00	3.02E-03	circBase	SO	5598
chr10:126370176-126370948-	4.92	8.99	3.23E-03	circBase	exonic	773
chr7:74171152-74172333+	4.92	8.98	3.26E-03	circBase	exonic	84
chr20:62407031-62422143-	4.91	8.99	3.30E-03	circBase	exonic	1255
chr16:30495148-30495584+	4.88	8.95	3.62E-03	circBase	exonic	284
chr6:31237743-31322442-	3.65	10.80	3.66E-03	novel	SO	84700
chr12:6619445-6619698+	4.84	8.95	3.89E-03	novel	intronic	254
chr3:114069121-114070725-	4.03	9.88	3.95E-03	circBase	exonic	1605
chr21:30693542-30702014+	4.78	8.91	4.49E-03	circBase	exonic	1836
chr14:73711352-73712883+	4.71	8.88	5.33E-03	Zhang2014	exonic	280
chr21:37619815-37620866+	4.69	8.87	5.56E-03	circBase	exonic	301
chr12:120995085-120995485+	4.63	8.83	6.46E-03	circBase	exonic	322
chr2:63206323-63223901+	4.62	8.82	6.72E-03	circBase	exonic	751
chr9:86293356-86301070-	4.61	8.82	6.80E-03	circBase	exonic	690
chr1:51868107-51874004-	4.60	8.81	6.96E-03	circBase	exonic	607
chr4:129913322-129925031-	3.69	9.68	7.31E-03	circBase	exonic	396
chr18:8076453-8088849+	4.51	8.76	8.83E-03	circBase	exonic	415
chr22:23243419-23261973+	4.50	8.77	8.84E-03	novel	intergenic	18555
chr16:30490412-30490782+	4.48	8.74	9.41E-03	novel	exonic	249
chr3:195686054-195686957-	4.47	8.74	9.67E-03	circBase	intronic	904
chr6:108984658-108986092+	4.46	8.73	1.00E-02	circBase	exonic	1435
chr19:50902108-50902741+	4.43	8.73	1.04E-02	circBase	exonic	317
chr5:49694941-49707217-	4.41	8.71	1.09E-02	circBase	SO	12277
chr4:73950966-73958017-	4.41	8.72	1.10E-02	circBase	exonic	1832
chr2:10559860-10560261+	4.41	8.71	1.12E-02	circBase	exonic	402
chr15:60734615-60737990-	4.39	8.71	1.14E-02	circBase	SO	3376
chr6:32487205-32549391-	4.38	8.69	1.18E-02	novel	SO	62187
chr7:5963018-5963593+	4.38	8.69	1.19E-02	circBase	exonic	287
chr19:45528587-45528995+	4.37	8.70	1.20E-02	circBase	exonic	224

chr7:129760589-129762042+	4.37	8.69	1.21E-02	circBase	exonic	304
chr22:47022648-47033857+	4.37	8.69	1.22E-02	circBase	exonic	332
chr10:13169745-13178897+	4.35	8.68	1.26E-02	circBase	SO	9153
chr9:110062422-110074018+	4.34	8.69	1.27E-02	circBase	exonic	487
chr13:100909849-100925600+	4.33	8.69	1.29E-02	circBase	exonic	428
chr1:41536267-41541123-	3.31	9.74	1.30E-02	circBase	exonic	360
chr21:38792601-38794168+	4.30	8.67	1.39E-02	circBase	exonic	179
chr5:72370569-72373320+	2.95	10.55	1.49E-02	circBase	exonic	268
chr5:137320946-137324004-	3.24	9.71	1.49E-02	circBase	exonic	331
chr4:36230204-36231267-	4.22	8.62	1.67E-02	circBase	exonic	1064
chr15:101906401-101910728-	4.20	8.62	1.72E-02	circBase	exonic	326
chr19:57967021-57967550-	4.20	8.61	1.73E-02	circBase	exonic	148
chr5:145144494-145205763-	4.17	8.60	1.85E-02	circBase	exonic	672
chr1:151139410-151139890+	4.16	8.59	1.89E-02	circBase	exonic	276
chr6:31238163-31323269-	2.86	10.20	1.91E-02	novel	SO	85107
chr22:23243156-23260373+	4.13	8.60	1.95E-02	novel	intergenic	17218
chr10:126370176-126384781-	4.09	8.58	2.13E-02	circBase	exonic	828
chr19:1032391-1032695+	4.09	8.57	2.15E-02	circBase	exonic	205
chr2:206992521-206994966-	4.09	8.57	2.17E-02	circBase	exonic	331
chr1:247318868-247323115-	2.86	10.09	2.17E-02	circBase	SO	4248
chr15:59323003-59323901+	4.09	8.57	2.17E-02	circBase	exonic	899
chr8:128902835-128903244+	4.09	8.56	2.21E-02	circBase	exonic	410
chr4:38055820-38104778+	4.07	8.56	2.27E-02	circBase	exonic	647
chr5:142416761-142437312+	4.07	8.55	2.28E-02	circBase	exonic	394
chr19:45901263-45901597-	4.07	8.56	2.28E-02	circBase	exonic	219
chr3:33725851-33738425-	4.07	8.55	2.28E-02	circBase	exonic	449
chr3:114412375-114436374-	4.04	8.54	2.41E-02	circBase	intronic	24000
chr6:51693821-51701267-	4.03	8.55	2.41E-02	novel	SO	7447
chr15:65471272-65472542-	4.04	8.54	2.42E-02	circBase	exonic	279
chr6:31239376-31324219+	3.06	9.56	2.45E-02	novel	SO	84844
chr3:119219542-119232566+	4.03	8.53	2.48E-02	circBase	exonic	402
chr3:149563798-149639014+	3.04	9.55	2.57E-02	circBase	exonic	716
chr3:119222379-119232566+	4.00	8.53	2.58E-02	circBase	exonic	236
chr19:17387304-17387718+	3.56	8.91	2.63E-02	circBase	exonic	217
chr2:34823138-34827426-	3.99	8.52	2.65E-02	novel	antisense	4289
chr15:32786219-32825569-	3.98	8.52	2.69E-02	novel	SO	39351
chr19:14568843-14569187+	3.98	8.52	2.73E-02	circBase	exonic	254
chr12:113705648-113707650+	3.98	8.51	2.73E-02	circBase	exonic	334
chr5:124036707-124036962-	2.80	9.83	2.74E-02	circBase	exonic	256
chrX:37245852-37285253+	3.97	8.51	2.77E-02	circBase	exonic	303
chr4:25848916-25849486-	3.96	8.52	2.79E-02	circBase	exonic	571
chr1:113196220-113202401+	3.96	8.51	2.79E-02	circBase	exonic	430
chr1:231930988-231954263+	3.96	8.51	2.81E-02	circBase	exonic	347

chr15:80390758-80415142+	3.96	8.51	2.83E-02	circBase	exonic	544
chr2:144966170-144969146-	3.96	8.50	2.86E-02	circBase	exonic	267
chr10:99196948-99197507-	3.94	8.50	2.91E-02	circBase	exonic	136
chr5:78936674-78964851+	3.93	8.51	2.93E-02	circBase	exonic	743
chr2:168920010-168931741-	3.42	8.99	2.96E-02	circBase	exonic	287
chr1:78183552-78191447-	3.93	8.49	3.01E-02	circBase	exonic	783
chr5:65284463-65290692+	3.22	9.07	3.04E-02	circBase	exonic	364
chr18:56363598-56367823+	3.92	8.49	3.08E-02	circBase	exonic	273
chr5:76342172-76344097+	3.90	8.48	3.19E-02	circBase	exonic	443
chr2:228881122-228884872-	3.88	8.48	3.25E-02	circBase	exonic	3751
chr1:233334685-233372726-	3.87	8.47	3.34E-02	circBase	exonic	844
chr14:78361811-78365065-	3.85	8.48	3.35E-02	novel	antisense	3255
chr10:125798031-125806240-	3.86	8.48	3.35E-02	circBase	exonic	1702
chr2:24046128-24046439-	3.86	8.48	3.36E-02	circBase	exonic	312
chr11:85685751-85695016-	3.86	8.48	3.37E-02	circBase	exonic	536
chr11:77330651-77336863-	3.86	8.47	3.40E-02	circBase	exonic	474
chr20:34317234-34320057-	3.36	8.93	3.40E-02	circBase	exonic	315
chr2:89082251-89092011+	3.86	8.46	3.45E-02	novel	exonic	615
chr5:56526673-56527148+	3.85	8.47	3.46E-02	circBase	exonic	348
chr4:148860976-148876525+	3.85	8.47	3.47E-02	circBase	exonic	222
chr9:88920107-88924932-	3.85	8.46	3.50E-02	circBase	exonic	508
chr15:90982564-90986710+	3.84	8.46	3.53E-02	circBase	exonic	446
chr20:13539655-13568017-	3.84	8.46	3.55E-02	circBase	exonic	393
chr2:37543380-37544322-	3.84	8.46	3.56E-02	circBase	exonic	943
chr19:57327951-57328316-	3.82	8.46	3.59E-02	novel	intronic	366
chr2:148653870-148657467+	3.03	9.20	3.60E-02	circBase	exonic	473
chr20:30954187-30959677+	3.41	8.83	3.62E-02	circBase	exonic	289
chr15:90984738-90986710+	3.82	8.45	3.67E-02	circBase	exonic	264
chr1:224553581-224559125+	3.82	8.45	3.68E-02	circBase	exonic	254
chr8:135521862-135533244-	3.80	8.45	3.75E-02	circBase	exonic	377
chr3:125567073-125567290-	3.81	8.45	3.76E-02	circBase	exonic	218
chr5:145197457-145205763-	3.12	9.01	3.76E-02	circBase	exonic	496
chr18:44526020-44526886+	3.81	8.45	3.77E-02	circBase	exonic	289
chr1:42730786-42776781-	3.80	8.45	3.82E-02	circBase	exonic	461
chr11:130130751-130131824-	2.61	9.79	3.86E-02	circBase	exonic	1074
chr6:155095123-155116273+	3.79	8.44	3.88E-02	circBase	exonic	576
chr12:95602619-95605043-	3.29	8.91	3.88E-02	circBase	exonic	2425
chr4:37633007-37640126-	2.66	9.70	3.92E-02	circBase	exonic	434
chr14:21698478-21702388+	3.77	8.45	3.94E-02	novel	antisense	3911
chr3:70448790-70449464-	3.77	8.45	3.94E-02	novel	intergenic	675
chr2:26486248-26500028+	3.78	8.44	3.95E-02	circBase	exonic	333
chr4:153332455-153333681-	2.38	10.39	4.03E-02	circBase	exonic	620
chr9:130206308-130207528+	3.77	8.43	4.07E-02	circBase	SO	1221

chr16:83940592-83945972+	3.76	8.44	4.07E-02	circBase	exonic	420
chr2:173435454-173460751+	3.75	8.43	4.17E-02	circBase	exonic	519
chr1:247322308-247323115-	3.75	8.42	4.20E-02	circBase	exonic	188
chr4:10099335-10105610-	3.75	8.42	4.21E-02	circBase	exonic	420
chr6:31239010-31324103-	2.15	13.00	4.27E-02	novel	SO	85094
chr17:1540003-1540356-	3.74	8.42	4.28E-02	circBase	exonic	269
chr14:21971316-21972024-	2.72	9.42	4.32E-02	circBase	exonic	623
chr20:21346059-21349228+	3.73	8.41	4.37E-02	circBase	exonic	329
chr4:151656410-151729550-	3.72	8.42	4.40E-02	circBase	exonic	449
chr19:45766371-45766625+	3.05	8.97	4.40E-02	circBase	exonic	103
chr12:116534474-116549317-	3.72	8.42	4.41E-02	circBase	exonic	169
chr2:169018297-169038600-	3.72	8.42	4.43E-02	circBase	exonic	420
chr19:11289021-11289396-	3.72	8.41	4.45E-02	circBase	exonic	295
chr15:44624186-44630515+	3.71	8.41	4.47E-02	circBase	exonic	317
chr10:95140976-95148911-	3.71	8.41	4.48E-02	circBase	exonic	420
chr1:42730786-42744343-	3.70	8.41	4.54E-02	circBase	exonic	400
chr7:91924203-91936970+	3.71	8.41	4.54E-02	circBase	exonic	576
chr10:7318854-7327916-	2.41	9.93	4.59E-02	circBase	exonic	434
chr19:18648411-18649246-	2.57	9.61	4.60E-02	circBase	exonic	394
chr14:99723808-99724173-	3.70	8.40	4.62E-02	circBase	exonic	366
chr2:114383428-114383979-	3.67	8.41	4.64E-02	novel	intronic	552
chr5:101624191-101624340-	3.67	8.41	4.64E-02	novel	intronic	150
chr15:80876391-80876848-	3.67	8.41	4.64E-02	novel	antisense	458
chr2:24787164-24807429+	3.69	8.40	4.70E-02	circBase	exonic	221
chr3:119222379-119236162+	3.69	8.40	4.72E-02	circBase	exonic	347
chr11:61133517-61135470+	3.67	8.40	4.77E-02	circBase	exonic	248
chr14:73614503-73614814+	3.67	8.40	4.78E-02	circBase	exonic	222
chr2:64083440-64085070+	3.67	8.40	4.80E-02	circBase	exonic	236
chr7:131071879-131084192+	3.67	8.39	4.84E-02	circBase	exonic	535
chr1:155874102-155874595-	3.66	8.40	4.88E-02	circBase	exonic	266
chr1:27056142-27059283+	2.99	8.95	4.88E-02	circBase	exonic	783
chr19:5604594-5604947-	2.63	9.36	4.90E-02	circBase	exonic	263
chr2:218388162-218391286-	3.65	8.40	4.91E-02	novel	intronic	3125
chr17:5268417-5276749+	3.65	8.40	4.91E-02	circBase	exonic	357
chr1:117944808-117984947+	2.44	9.77	4.94E-02	circBase	exonic	648
chr3:171969050-172016577+	3.65	8.39	4.94E-02	circBase	exonic	553
chr1:26772807-26774151+	3.66	8.39	4.94E-02	circBase	exonic	219
chr2:63660879-63667005-	3.66	8.39	4.94E-02	circBase	exonic	441
chr12:28458582-28460682+	3.65	8.39	4.98E-02	circBase	exonic	467
chrM:14068-14923+	-7.99	10.32	3.97E-12	novel	SO	856
chrM:14131-15754+	-6.37	10.30	5.35E-09	novel	SO	1624
chrM:7749-8685-	-6.54	9.24	5.83E-08	novel	SO	937
chrM:4198-6296+	-6.46	9.20	9.26E-08	novel	SO	2099

chrM:14068-14413+	-5.59	10.19	1.11E-07	novel	SO	346
chrM:14131-15754-	-5.25	10.96	1.47E-07	novel	SO	1624
chrM:13856-14217-	-6.25	9.07	3.70E-07	novel	intronic	362
chrM:14056-14263+	-5.73	9.58	3.98E-07	novel	SO	208
chrM:14056-14263-	-5.01	10.50	6.85E-07	novel	intronic	208
chrM:14068-14827+	-6.11	8.98	9.39E-07	novel	SO	760
chrM:14414-14923-	-5.48	9.64	1.09E-06	novel	intronic	510
chrM:14068-14923-	-4.66	11.05	1.59E-06	novel	intronic	856
chrM:13856-14421-	-6.02	8.93	1.64E-06	novel	intronic	566
chrM:14056-14377+	-4.26	11.38	6.74E-06	novel	SO	322
chrM:14074-14394-	-5.70	8.77	1.09E-05	novel	intronic	321
chrM:14213-15778+	-5.70	8.76	1.13E-05	novel	SO	1566
chrM:7749-8685+	-5.48	8.66	3.86E-05	novel	SO	937
chrM:14068-14446-	-4.98	9.05	4.64E-05	novel	intronic	379
chrM:14068-14413-	-3.82	11.23	4.81E-05	novel	intronic	346
chrM:13861-15754-	-5.41	8.63	5.72E-05	novel	SO	1894
chrM:13940-15335-	-5.40	8.62	6.18E-05	novel	intronic	1396
chrM:14068-15382+	-5.39	8.62	6.40E-05	novel	SO	1315
chrM:14056-14377-	-3.47	12.23	1.32E-04	novel	intronic	322
chrM:14068-15382-	-4.56	9.07	1.46E-04	novel	intronic	1315
chrM:5483-6492-	-4.71	8.98	1.51E-04	novel	SO	1010
chrM:14213-15778-	-4.41	9.14	1.68E-04	novel	SO	1566
chrM:14056-16359-	-5.12	8.51	2.41E-04	novel	SO	2304
chrM:14068-14446+	-4.13	9.12	4.30E-04	novel	SO	379
chrM:13847-14413-	-4.20	9.04	4.70E-04	novel	intronic	567
chrM:10509-12709-	-4.83	8.40	9.15E-04	novel	intronic	2201
chr7:61968974-61969313-	-3.42	9.67	1.02E-03	novel	intergenic	340
chr4:49287884-49293902+	-4.79	8.38	1.10E-03	novel	intergenic	6019
chrM:4198-6296-	-3.45	9.63	1.16E-03	novel	SO	2099
chrM:5169-8175-	-4.72	8.36	1.45E-03	novel	SO	3007
chr10:116879949-116931050+	-4.71	8.36	1.50E-03	circBase	exonic	1055
chr7:2472529-2472892+	-4.70	8.35	1.61E-03	novel	SO	364
chrM:13940-15335+	-4.68	8.35	1.72E-03	novel	SO	1396
chrM:5483-6492+	-4.60	8.32	2.39E-03	novel	SO	1010
chr7:45351009-45351463+	-4.58	8.31	2.62E-03	novel	intergenic	455
chrM:13847-14413+	-4.54	8.30	3.14E-03	novel	SO	567
chrM:9191-9761-	-4.52	8.30	3.36E-03	novel	intronic	571
chr10:116879949-116889297+	-3.73	8.80	3.49E-03	circBase	exonic	536
chrM:14068-14827-	-4.50	8.29	3.62E-03	novel	intronic	760
chrM:14056-15335+	-4.49	8.29	3.78E-03	novel	SO	1280
chr1:45925067-45925864-	-4.49	8.29	3.91E-03	novel	intronic	798
chr4:49313820-49319959-	-3.47	8.91	4.60E-03	novel	intronic	6140
chr3:63141694-63146475+	-4.40	8.26	5.68E-03	novel	intergenic	4782

chrM:5338-9082-	-4.39	8.26	5.70E-03	novel	SO	3745
chrM:5169-8175+	-4.38	8.25	6.05E-03	novel	SO	3007
chrM:8928-12907+	-4.37	8.25	6.16E-03	novel	SO	3980
chr17:76840825-76841186+	-4.37	8.25	6.38E-03	novel	intergenic	362
chrX:153674763-153677645+	-4.34	8.24	7.25E-03	novel	exonic	429
chrM:14391-15754-	-4.32	8.24	7.78E-03	novel	SO	1364
chrM:6337-9004-	-4.29	8.23	8.45E-03	novel	SO	2668
chr4:115540579-115544858+	-3.97	8.44	8.98E-03	circBase	exonic	4280
chrM:5169-6247+	-2.80	9.39	9.36E-03	novel	SO	1079
chr12:51858305-51867619-	-4.27	8.22	9.49E-03	novel	antisense	9315
chr5:101612950-101613137-	-4.27	8.22	9.49E-03	novel	intronic	188
chrM:14131-15286-	-4.27	8.22	9.49E-03	novel	intronic	1156
chrM:13856-15749-	-4.26	8.22	9.80E-03	novel	SO	1894
chrM:10905-13384-	-4.25	8.22	9.90E-03	novel	intronic	2480
chrM:14056-14286-	-4.25	8.22	9.95E-03	novel	intronic	231
chrM:1696-4992-	-4.25	8.22	9.99E-03	novel	SO	3297
chrM:6358-6603-	-4.25	8.22	1.01E-02	novel	antisense	246
chr3:177365664-177366349-	-4.24	8.21	1.06E-02	novel	antisense	686
chr5:9890487-9891713-	-4.24	8.21	1.06E-02	novel	intronic	1227
chrM:5426-5929+	-4.24	8.21	1.06E-02	novel	SO	504
chrM:497-1716+	-3.59	8.58	1.07E-02	novel	SO	1220
chrM:3508-5482-	-4.23	8.21	1.09E-02	novel	SO	1975
chr1:156641522-156641776-	-4.22	8.21	1.11E-02	novel	SO	255
chr14:61764146-61765213+	-4.22	8.21	1.11E-02	novel	intronic	1068
chr2:7098168-7099459-	-4.22	8.21	1.11E-02	novel	antisense	1292
chrM:12550-13384+	-4.22	8.21	1.12E-02	novel	SO	835
chr19:15451480-15451957+	-4.19	8.20	1.25E-02	novel	intergenic	478
chr18:33027680-33029121+	-4.19	8.20	1.25E-02	novel	antisense	1442
chr8:124691024-124692645-	-4.19	8.20	1.25E-02	novel	intergenic	1622
chr1:237452272-237452648+	-4.18	8.20	1.32E-02	novel	intronic	377
chrM:14414-14923+	-3.42	8.64	1.32E-02	novel	SO	510
chrM:2227-10629-	-4.16	8.19	1.42E-02	novel	SO	8403
chrM:4656-4839-	-4.13	8.19	1.52E-02	novel	antisense	184
chr6:7203706-7203883+	-4.12	8.19	1.61E-02	novel	intronic	178
chr8:108602811-108603110+	-4.12	8.19	1.61E-02	novel	intergenic	300
chr12:51858305-51867619+	-4.12	8.19	1.61E-02	novel	SO	9315
chr2:1863526-1863933-	-4.12	8.19	1.61E-02	novel	intronic	408
chr1:152190972-152192999-	-4.12	8.19	1.61E-02	novel	SO	2028
chr21:27864014-27865290-	-4.12	8.19	1.61E-02	novel	intronic	1277
chrM:14068-15403-	-3.01	8.84	1.67E-02	novel	intronic	1336
chrM:5131-5369+	-4.11	8.18	1.70E-02	novel	SO	239
chr1:44782910-44783102+	-4.10	8.18	1.72E-02	novel	antisense	193
chr2:191765290-191769893+	-4.09	8.18	1.75E-02	circBase	exonic	374

chrM:11046-13436-	-4.09	8.18	1.77E-02	novel	intronic	2391
chrM:2227-8822-	-4.09	8.18	1.77E-02	novel	SO	6596
chr4:72385598-72386636+	-4.09	8.18	1.77E-02	novel	intronic	1039
chr5:99662584-99662955+	-4.09	8.18	1.77E-02	novel	intergenic	372
chr10:79015294-79015606-	-4.08	8.18	1.80E-02	novel	intronic	313
chrM:5112-5369+	-4.09	8.18	1.81E-02	novel	SO	258
chr1:175329032-175329668-	-4.09	8.18	1.81E-02	novel	intronic	637
chr4:129388873-129390492-	-4.08	8.17	1.85E-02	novel	antisense	1620
chr12:108382221-108382505-	-4.08	8.17	1.85E-02	novel	intergenic	285
chrM:3502-4499-	-3.44	8.51	1.86E-02	novel	SO	998
chrM:4108-5161-	-4.05	8.17	2.00E-02	novel	SO	1054
chr6:132693747-132695916-	-4.05	8.17	2.01E-02	circBase	exonic	399
chrM:14808-15740-	-4.04	8.16	2.08E-02	novel	SO	933
chrM:3323-3598-	-4.03	8.16	2.13E-02	novel	antisense	276
chr1:91683557-91686825-	-4.03	8.16	2.14E-02	novel	intergenic	3269
chr19:3381710-3435205+	-4.03	8.16	2.14E-02	novel	exonic	928
chr11:110420168-110421222+	-4.03	8.16	2.14E-02	novel	intergenic	1055
chrX:149807416-149832082+	-4.03	8.16	2.14E-02	circBase	exonic	1200
chrM:15336-15743-	-4.03	8.16	2.14E-02	novel	SO	408
chr20:44305609-44306725+	-4.02	8.16	2.16E-02	novel	intergenic	1117
chr15:64814029-64814893+	-4.00	8.15	2.32E-02	novel	intronic	865
chrY:2316270-2338639-	-3.98	8.15	2.46E-02	novel	intronic	22370
chr3:133613862-133614703-	-3.97	8.15	2.49E-02	novel	SO	842
chr8:53280083-53281440+	-3.97	8.15	2.49E-02	novel	antisense	1358
chrM:8777-9082-	-3.96	8.15	2.62E-02	novel	intronic	306
chr14:97986485-97986730-	-3.96	8.15	2.62E-02	novel	antisense	246
chr10:92812462-92813044+	-3.96	8.15	2.62E-02	novel	intronic	583
chr1:91296561-91296993+	-3.96	8.15	2.62E-02	novel	antisense	433
chrX:115089838-115090473-	-3.96	8.15	2.62E-02	novel	intergenic	636
chr5:116439683-116440980-	-3.96	8.15	2.62E-02	novel	intergenic	1298
chr22:43624858-43625077-	-3.96	8.15	2.62E-02	novel	intronic	220
chr1:244361261-244361599+	-3.96	8.15	2.62E-02	novel	intergenic	339
chrM:3502-4499+	-3.96	8.15	2.64E-02	novel	SO	998
chrM:14005-14421-	-3.95	8.14	2.66E-02	novel	intronic	417
chr4:129689520-129689798-	-3.96	8.15	2.67E-02	novel	intergenic	279
chr18:2912577-2913396-	-3.95	8.14	2.73E-02	novel	antisense	820
chrM:13856-14421+	-3.95	8.14	2.73E-02	novel	SO	566
chr7:117466146-117468267-	-3.93	8.14	2.85E-02	novel	intronic	2122
chr1:9908665-9908967-	-3.93	8.14	2.85E-02	novel	SO	303
chr17:1714257-1714431-	-3.93	8.14	2.85E-02	novel	intronic	175
chr1:248669490-248669819-	-3.93	8.14	2.85E-02	novel	intergenic	330
chr3:43678425-43678728-	-3.93	8.14	2.85E-02	novel	intronic	304
chr5:140708075-140708474+	-3.93	8.14	2.85E-02	novel	SO	400

chr10:8253653-8254219-	-3.93	8.14	2.85E-02	novel	intergenic	567
chr11:18307947-18310474-	-3.93	8.14	2.85E-02	novel	SO	2528
chr2:6800477-6800704-	-3.93	8.14	2.85E-02	novel	intergenic	228
chr18:14501880-14503789+	-3.93	8.14	2.85E-02	novel	SO	1910
chr9:139784160-139786287+	-3.93	8.14	2.85E-02	novel	intronic	2128
chr5:13469962-13470695-	-3.93	8.14	2.87E-02	novel	intergenic	734
chr5:169822309-169822680+	-3.92	8.14	2.93E-02	novel	intronic	372
chrM:5112-5369-	-3.92	8.14	2.94E-02	novel	antisense	258
chr16:17645261-17646025+	-3.92	8.14	2.95E-02	novel	intergenic	765
chr15:96435235-96435957-	-3.92	8.14	2.95E-02	novel	intergenic	723
chr2:196901572-196901759-	-3.92	8.14	2.95E-02	novel	intronic	188
chr2:7098168-7099459+	-3.92	8.14	2.95E-02	novel	intronic	1292
chr11:127075650-127075930-	-3.92	8.14	2.95E-02	novel	antisense	281
chr19:45985744-45986030+	-3.92	8.14	2.95E-02	novel	intergenic	287
chrX:58105171-58105736+	-3.92	8.14	2.95E-02	novel	intergenic	566
chr7:95559307-95559643-	-3.92	8.14	2.95E-02	novel	antisense	337
chr7:57685162-57686007+	-3.92	8.14	2.95E-02	novel	intergenic	846
chr2:125593784-125594801-	-3.92	8.14	2.95E-02	novel	antisense	1018
chr15:92357598-92357976+	-3.92	8.14	2.95E-02	novel	intergenic	379
chr13:50454666-50454890-	-3.92	8.14	2.95E-02	novel	intergenic	225
chr2:42799963-42801369+	-3.92	8.14	2.95E-02	novel	intronic	1407
chr1:222889026-222895856+	-3.90	8.13	3.08E-02	circBase	exonic	417
chrM:15492-15740-	-2.33	9.36	3.13E-02	novel	SO	249
chr4:49236069-49237123-	-3.89	8.13	3.18E-02	novel	SO	1055
chr15:54304845-54308083+	-3.19	8.47	3.35E-02	circBase	exonic	3239
chr1:202569428-202569917+	-3.86	8.12	3.43E-02	novel	antisense	490
chr8:47460250-47461040-	-3.86	8.12	3.43E-02	novel	antisense	791
chr6:136958463-137019820-	-3.85	8.12	3.52E-02	circBase	exonic	1404
chrX:43761657-43761834-	-3.85	8.12	3.52E-02	novel	intergenic	178
chr17:64119383-64179425+	-3.85	8.12	3.52E-02	novel	antisense	60043
chr1:27241767-27241990+	-3.85	8.12	3.52E-02	novel	intronic	224
chr3:41973336-41979689-	-3.85	8.12	3.52E-02	circBase	exonic	403
chr16:15832422-15839094-	-3.85	8.12	3.52E-02	novel	exonic	710
chr16:49138902-49139448+	-3.85	8.12	3.52E-02	novel	intergenic	547
chr12:52844213-52885328-	-3.82	8.12	3.80E-02	novel	SO	41116
chr6:1856607-1856949+	-3.82	8.12	3.81E-02	novel	antisense	343
chrX:118774655-118787003-	-3.81	8.11	3.87E-02	circBase	exonic	446
chr14:61936099-61936956+	-3.81	8.11	3.91E-02	novel	intronic	858
chr3:136183723-136221621-	-3.63	8.26	3.93E-02	circBase	exonic	637
chr12:100551829-100552849+	-3.79	8.11	4.06E-02	novel	antisense	1021
chr14:73299114-73299693+	-3.79	8.11	4.08E-02	novel	antisense	580
chr19:28266541-28267062-	-3.79	8.11	4.09E-02	novel	intronic	522
chr3:123796534-123797538+	-3.78	8.11	4.19E-02	novel	intronic	1005

chr19:28553203-28553507+	-3.78	8.11	4.19E-02	novel	intergenic	305
chr3:12710399-12713300-	-3.78	8.11	4.19E-02	novel	intergenic	2902
chr15:38771791-38773746+	-3.78	8.11	4.19E-02	novel	intronic	1956
chr1:109666661-109667928+	-3.78	8.11	4.19E-02	novel	intronic	1268
chr10:85323694-85324204-	-3.78	8.11	4.19E-02	novel	intergenic	511
chr2:30936325-30937849-	-3.78	8.11	4.19E-02	novel	intergenic	1525
chr2:99226033-99230401-	-3.78	8.11	4.19E-02	novel	antisense	4369
chr15:69406188-69407439+	-3.78	8.11	4.19E-02	novel	intronic	1252
chr8:19905289-19905608+	-3.78	8.11	4.19E-02	novel	intergenic	320
chr22:19356355-19358437-	-3.78	8.11	4.19E-02	novel	intronic	2083
chrM:1717-6042-	-3.78	8.11	4.19E-02	novel	SO	4326
chr1:34410581-34411217-	-3.78	8.11	4.19E-02	novel	intronic	637
chr19:9009600-9021128+	-3.78	8.11	4.19E-02	novel	antisense	11529
chr6:29435933-29436414-	-3.78	8.11	4.19E-02	novel	intronic	482
chr19:56268865-56278866+	-3.78	8.11	4.19E-02	novel	SO	10002
chr5:15450759-15451641+	-3.78	8.11	4.19E-02	novel	SO	883
chr12:77738253-77738994-	-3.78	8.11	4.19E-02	novel	antisense	742
chr3:28378920-28379198-	-3.78	8.11	4.19E-02	novel	intronic	279
chr2:217463863-217465967-	-3.78	8.11	4.19E-02	novel	intronic	2105
chr9:17714171-17715298+	-3.78	8.11	4.19E-02	novel	intronic	1128
chr18:39330936-39331282+	-3.78	8.11	4.19E-02	novel	intergenic	347
chr5:64084778-64100213+	-3.78	8.11	4.21E-02	circBase	exonic	339
chr15:66044717-66048810-	-3.78	8.11	4.21E-02	circBase	exonic	583
chr17:41135771-41136994+	-3.78	8.11	4.22E-02	novel	intronic	1224
chr16:52635508-52635756+	-3.78	8.11	4.23E-02	novel	antisense	249
chrM:8686-9082+	-3.78	8.11	4.23E-02	novel	SO	397
chrM:14530-15382-	-3.76	8.10	4.45E-02	novel	intronic	853
chrM:8662-12964-	-3.76	8.10	4.46E-02	novel	intronic	4303
chr5:1157475-1157862+	-3.76	8.10	4.50E-02	novel	intergenic	388
chr7:35421329-35424241-	-3.76	8.10	4.50E-02	novel	intergenic	2913
chr3:3608192-3608637-	-3.76	8.10	4.50E-02	novel	intronic	446
chr6:110919137-110919620+	-3.76	8.10	4.50E-02	novel	SO	484
chr16:66831411-66833401+	-3.76	8.10	4.50E-02	novel	antisense	1991
chr14:35572819-35573501-	-3.76	8.10	4.50E-02	novel	intronic	683
chr1:10913349-10913929-	-3.76	8.10	4.50E-02	novel	intergenic	581
chr5:52701772-52701939-	-3.76	8.10	4.50E-02	novel	intergenic	168
chr17:72677891-72678049-	-3.76	8.10	4.50E-02	novel	antisense	159
chr5:128951199-128951741+	-3.76	8.10	4.50E-02	novel	intronic	543
chr18:70821613-70822484-	-3.76	8.10	4.50E-02	novel	intronic	872
chrM:10949-11578+	-3.76	8.10	4.50E-02	novel	SO	630
chr1:179134240-179134528+	-3.76	8.10	4.50E-02	novel	antisense	289
chr1:47457320-47457923-	-3.76	8.10	4.50E-02	novel	antisense	604
chr16:25537610-25537942-	-3.76	8.10	4.50E-02	novel	intergenic	333

chr16:30778048-30778233-	-3.76	8.10	4.50E-02	novel	antisense	186
chr6:26729788-26772320-	-3.76	8.10	4.50E-02	novel	SO	42533
chr19:53093436-53095956+	-3.76	8.10	4.50E-02	novel	SO	2521
chr19:49889243-49891036-	-3.76	8.10	4.50E-02	novel	SO	1794
chr16:30862650-30863900+	-3.76	8.10	4.50E-02	novel	antisense	1251
chr5:99725917-99727554-	-3.76	8.10	4.50E-02	novel	antisense	1638
chr7:132268918-132270784-	-3.76	8.10	4.50E-02	novel	intronic	1867
chr10:94384776-94386767+	-3.76	8.10	4.50E-02	novel	intronic	1992
chr8:140615413-140615846-	-3.76	8.10	4.50E-02	novel	intronic	434
chr3:145220990-145222384-	-3.76	8.10	4.50E-02	novel	intergenic	1395
chrX:104694053-104694338+	-3.76	8.10	4.50E-02	novel	intronic	286
chr16:6236410-6236658-	-3.76	8.10	4.50E-02	novel	antisense	249
chr17:72677878-72678036-	-3.76	8.10	4.50E-02	novel	antisense	159
chr16:22297515-22298463-	-3.76	8.10	4.50E-02	novel	SO	949
chr6:4665244-4665859-	-3.76	8.10	4.50E-02	novel	intergenic	616
chr7:155066222-155067481-	-3.76	8.10	4.50E-02	novel	intronic	1260
chr1:206654794-206655594+	-3.76	8.10	4.50E-02	novel	intronic	801
chr4:10120654-10120999+	-3.75	8.10	4.52E-02	novel	intergenic	346
chr6:157150361-157222659+	-3.74	8.10	4.57E-02	circBase	exonic	345
chrM:4393-5328+	-3.75	8.10	4.58E-02	novel	SO	936
chrX:11066628-11067116+	-3.74	8.10	4.64E-02	novel	antisense	489
chr2:117077226-117077653+	-3.74	8.10	4.64E-02	novel	intergenic	428
chr5:19653910-19654298+	-3.74	8.10	4.64E-02	novel	antisense	389
chr1:33797747-33798148-	-3.74	8.10	4.64E-02	novel	SO	402
chr11:118757228-118757501-	-3.74	8.10	4.64E-02	novel	SO	274
chr8:1595153-1597157+	-3.74	8.10	4.64E-02	novel	intronic	2005
chr3:100967695-100968066-	-3.74	8.10	4.64E-02	novel	intronic	372
chr15:92357598-92357976-	-3.74	8.10	4.64E-02	novel	intergenic	379
chr1:38191627-38193092-	-3.74	8.10	4.64E-02	novel	SO	1466
chr1:245251280-245252960+	-3.74	8.10	4.64E-02	novel	intronic	1681
chr13:42859830-42860435+	-3.74	8.10	4.64E-02	novel	SO	606
chr1:247687539-247687776-	-3.74	8.10	4.64E-02	novel	antisense	238
chr2:242881954-242882185-	-3.74	8.10	4.64E-02	novel	antisense	232
chrX:134743064-134744999-	-3.74	8.10	4.64E-02	novel	antisense	1936
chr3:183927773-183928090+	-3.74	8.10	4.64E-02	novel	intronic	318
chr16:17569732-17570006-	-3.74	8.10	4.64E-02	novel	intergenic	275
chr20:8172717-8173554-	-3.74	8.10	4.64E-02	novel	antisense	838
chr11:128084724-128084928+	-3.74	8.10	4.64E-02	novel	intronic	205
chr5:2473864-2474391-	-3.74	8.10	4.64E-02	novel	intergenic	528
chr5:3872006-3872524-	-3.74	8.10	4.64E-02	novel	intergenic	519
chr1:107752658-107753123-	-3.74	8.10	4.64E-02	novel	antisense	466
chrX:36876690-36876915-	-3.74	8.10	4.64E-02	novel	intergenic	226
chrX:41253998-41254311+	-3.74	8.10	4.64E-02	novel	intergenic	314

chr12:11719773-11722273+	-3.74	8.10	4.64E-02	novel	intronic	2501
chr1:756025-757700-	-3.74	8.10	4.64E-02	novel	antisense	1676
chr19:21067189-21069812+	-3.74	8.10	4.64E-02	novel	intergenic	2624
chr17:7452936-7453120+	-3.74	8.10	4.64E-02	novel	intronic	185
chr7:144063319-144064150+	-3.74	8.10	4.64E-02	novel	SO	832
chr13:24333434-24334356-	-3.74	8.10	4.64E-02	novel	SO	923
chr21:23363744-23363981+	-3.74	8.10	4.64E-02	novel	intergenic	238
chr1:22212288-22213055-	-3.74	8.10	4.64E-02	novel	intronic	768
chr4:4772121-4774202-	-3.74	8.10	4.64E-02	novel	antisense	2082
chr14:95980593-95982253-	-3.74	8.10	4.64E-02	novel	intergenic	1661
chr9:93145629-93146544-	-3.74	8.10	4.66E-02	novel	SO	916
chr17:80049597-80050326+	-3.73	8.10	4.69E-02	novel	antisense	730
chrM:14074-14394+	-3.73	8.10	4.74E-02	novel	SO	321
chrM:8482-9043+	-3.72	8.10	4.79E-02	novel	SO	562
chr1:156464351-156464588+	-3.71	8.09	4.97E-02	novel	antisense	238

logFC=$\log_2$ fold change. logCPM=$\log_2$CPM by edgeR. PValue=*P*-value calculated by edgeR. length=predicted length of circRNAs. SO=sense overlapping circRNAs. Zhang2014=identified by Zhang et al, 2014 (1).

Supplemental Table 3. Primers of the candidate circRNAs.

**circRNA**	**circBase ID**	**Direction**	**Primer**
chr9:37424842-37426651+	hsa_circ_0001861	F	CAGATACCACCGCCGAAC
chr9:37424842-37426651+	hsa_circ_0001861	R	CATCCGAGTCCCACTGCT
chr10:5836848-5842668-	hsa_circ_0002665	F	CTGAAGCAGAAGCCCTGG
chr10:5836848-5842668-	hsa_circ_0002665	R	CGGGTGGTGATCCTGGTA
chr21:30693542-30702014+	hsa_circ_0001181	F	AGCGCTGTCGCAAGAGAA
chr21:30693542-30702014+	hsa_circ_0001181	R	TCCGCTGGTCATTAAGGC
chr19:13039156-13039661-	hsa_circ_0000896	F	TCCTGGAGATGGGGTTCA
chr19:13039156-13039661-	hsa_circ_0000896	R	GCCAAGAGCCACTGGAGA
chr21:16386665-16415895-	hsa_circ_0004771	F	GCAGAGGCTCAGAGCTTGG
chr21:16386665-16415895-	hsa_circ_0004771	R	TGGCTGTGTTTCTCCCAAA
chr7:129760589-129762042+	hsa_circ_0002190	F	GCCATCATCAATGGCTCC
chr7:129760589-129762042+	hsa_circ_0002190	R	GCCGTTGCTCTCTCCAAA
chr1:41536267-41541123-	hsa_circ_0000061	F	CCTGGCAGCAAGAGGAAA
chr1:41536267-41541123-	hsa_circ_0000061	R	CGGAGGCAGGATAGGGAT
chr3:114069121-114070725-	hsa_circ_0005332	F	CACAGCCAGTGGGCAAG
chr3:114069121-114070725-	hsa_circ_0005332	R	CTCGAGCACGGAATTGCT
chr7:5963018-5963593+	hsa_circ_0007177	F	GATCGGCGGGAGCTCTAT
chr7:5963018-5963593+	hsa_circ_0007177	R	TGGGCGTTTTCCTCATGT
chr15:80390758-80415142+	hsa_circ_0000642	F	CATCTATGCAGCCCAGCC

chr15:80390758-80415142+	hsa_circ_0000642	R	CATTAGCTTTCTGCCTTCCC
chr4:129913322-129925031-	hsa_circ_0001439	F	TGTGATAATCGAACAACTCCG
chr4:129913322-129925031-	hsa_circ_0001439	R	AGGGGAAAGGCCTCCAAT
chr5:137320946-137324004-	hsa_circ_0001535	F	GACTGTTCAAAACCTGTGGCT
chr5:137320946-137324004-	hsa_circ_0001535	R	TGGTAGGATGCTGATGGGA
chr4:73950966-73958017-	hsa_circ_0001417	F	CACCCGCAGCTGCTAACT
chr4:73950966-73958017-	hsa_circ_0001417	R	TGGTAGGTGCTGATGACCC
chr1:51868107-51874004-	hsa_circ_0005567	F	CGCTGACAGGTCCAGTTG
chr1:51868107-51874004-	hsa_circ_0005567	R	TGGAGATCTGCGATTCCTG
chr19:45528587-45528995+	hsa_circ_0008590	F	ACCAGCAGGGACAGATGC
chr19:45528587-45528995+	hsa_circ_0008590	R	TGGTTCTTCAGGGACCCA
chr2:63206323-63223901+	hsa_circ_0005552	F	AGCGCCTTCGCTATCTCA
chr2:63206323-63223901+	hsa_circ_0005552	R	TCTGCAGCCATTTCACCA
chr21:37619815-37620866+	hsa_circ_0001187	F	CACCATGGCCCTTCTCTG
chr21:37619815-37620866+	hsa_circ_0001187	R	CCTGCAGCCATGGTCTTT
chr2:148653870-148657467+	hsa_circ_0001073	F	TTGCGGGGATTGTCATTT
chr2:148653870-148657467+	hsa_circ_0001073	R	AAACAATGCCGCCGTTTA
chr12:120995085-120995485+	hsa_circ_0028899	F	GGGAGAAAGGGGTGTTGG
chr12:120995085-120995485+	hsa_circ_0028899	R	GCATGCCCAGCAGAAGAT
chr10:126370176-126370948-	hsa_circ_0000267	F	CCCAGCCGTGTGTCCTTA
chr10:126370176-126370948-	hsa_circ_0000267	R	GTCCTTTTCAGGCAGGCA

F=forward. R=reverse. circRNA=circular RNA.

Supplemental Table 4. The information of the candidate circRNAs

circBaseID	chrom	start	end	strand	Threshold
hsa_circ_0002665	chr10	5836847	5842668	-	0.080158
hsa_circ_0001181	chr21	30693541	30702014	+	0.183668
hsa_circ_0000896	chr19	13039155	13039661	-	0.091827
hsa_circ_0004771	chr21	16386664	16415895	-	3.129296
hsa_circ_0002190	chr7	129760588	129762042	+	0.034171
hsa_circ_0000061	chr1	41536266	41541123	-	0.105503
hsa_circ_0005332	chr3	114069120	114070725	-	0.052197
hsa_circ_0007177	chr7	5963017	5963593	+	0.298217
hsa_circ_0000642	chr15	80390757	80415142	+	0.451966
hsa_circ_0001439	chr4	129913321	129925031	-	1.19933
hsa_circ_0001535	chr5	137320945	137324004	-	4.37801
hsa_circ_0001417	chr4	73950965	73958017	-	0.362562
hsa_circ_0005567	chr1	51868106	51874004	-	0.642511
hsa_circ_0005552	chr2	63206322	63223901	+	0.135137
hsa_circ_0001187	chr21	37619814	37620866	+	0.070941
hsa_circ_0001073	chr2	148653869	148657467	+	0.0657
hsa_circ_0028899	chr12	120995084	120995485	+	2.316445
hsa_circ_0000267	chr10	126370175	126370948	-	0.921607

hsa_circ_0006404	chr6	108984657	108986092	+	0.365311
hsa_circ_0000690	chr16	30495147	30495584	+	0.663593

Chrom=chromosome. Start=the start genomic position of circRNAs. End= the end genomic position of circRNAs. Threshold=threshold for discretization.

Supplementary Figure 1. The relative levels of 20 selected circRNAs

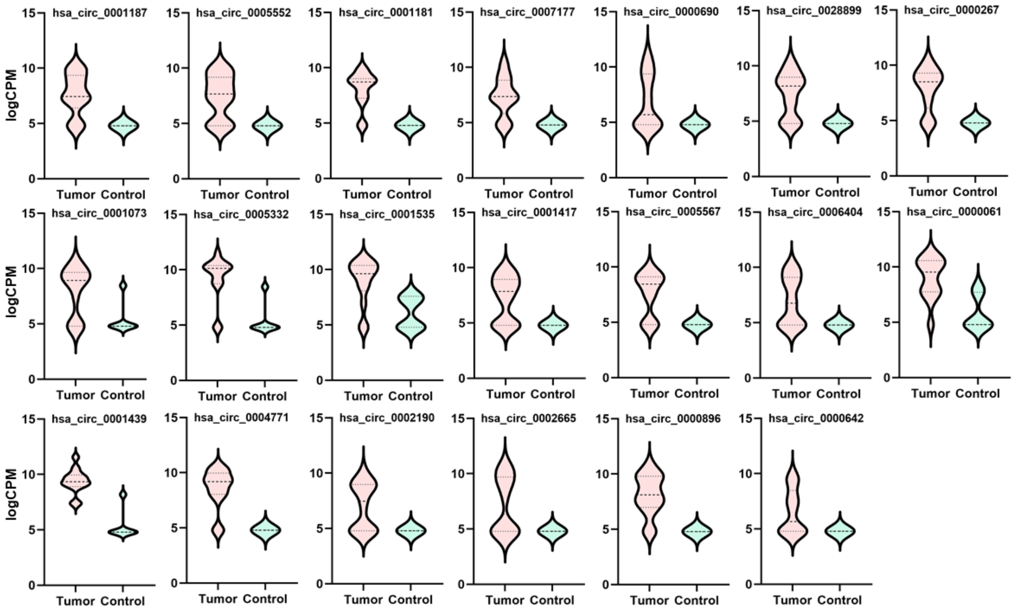

CPM value was calculated by edgR.

Data repositories

The aligned reads of the merged samples reported in this paper have been deposited at the database of NODE (https://www.biosino.org/node, project ID: OEP001254).

Reference

1. Zhang, Z., Qi, S., Tang, N., Zhang, X., Chen, S., Zhu, P., Ma, L., Cheng, J., Xu, Y., Lu, M. *et al.* (2014) Discovery of replicating circular RNAs by RNA-seq and computational algorithms. *PLoS Pathog*, 10, e1004553.
